# No causal link between estradiol and Alzheimer's disease in females: a Mendelian randomization study

**DOI:** 10.1002/alz70855_101658

**Published:** 2025-12-23

**Authors:** Claudia Barth

**Affiliations:** ^1^ Diakonhjemmet Hospital, Oslo, Oslo, Norway

## Abstract

**Background:**

Estradiol – the most potent estrogen in females – has been implicated in the development of Alzheimer's disease (AD) – a sex‐biased brain disorder with a female preponderance. Yet, while some studies suggest protective effects of estradiol on AD risk, results are inconsistent partly due to methodological challenges of observational studies such as confounding and reverse causation. Mendelian randomization (MR) can overcome these limitations by using genetic variants to test for causal associations. Here, we examined whether genetically predicted estradiol‐related factors (exposures) are causally linked to AD risk (outcome) in females, by conducting two‐sample MR analyses.

**Method:**

Two‐sample MR analyses were conducted using female‐specific summary statistics from previously conducted and newly run genome‐wide association studies (GWAS). We ran and annotated GWAS on sex‐specific estradiol levels, both in females and males, using data from the UK Biobank. Exposure variables included estradiol levels as well as factors related to lifetime estradiol exposure (i.e., reproductive span, age at menarche, age at menopause, and number of childbirths). For the univariable MR analyses, we applied the inverse‐variance weighted method and robust estimation methods including MR‐Egger, weighted median, simple mode, and weighted mode. To ensure the robustness of results, we run several sensitivity analyses such as including BMI as confounder in multivariable MR analyses and replicated our estradiol findings in a stratified pre‐and postmenopausal UK Biobank sample, an independent sample of females from the LIFE‐Adult and LIFE‐Heart study as well as in a male‐only UK Biobank sample.

**Result:**

Across all female‐only samples, we consistently did not find any significant associations between estradiol‐related factors and AD risk. These results were robust throughout sensitivity analyses and using a male‐only sample.

**Conclusion:**

The results do not support a causal link of estradiol‐related factors and AD risk in females as well as males. Future studies should assess other influencing factors, as well as implement sex‐specific, causal frameworks to examine potential time‐varying effects of hormonal fluctuations and periods of increased susceptibility to sex steroids such as the perimenopausal period.

